# Gate‐Tunable Magnetism via Resonant Se‐Vacancy Levels in WSe_2_

**DOI:** 10.1002/advs.202102911

**Published:** 2021-10-28

**Authors:** Tuan Dung Nguyen, Jinbao Jiang, Bumsub Song, Minh Dao Tran, Wooseon Choi, Ji Hee Kim, Young‐Min Kim, Dinh Loc Duong, Young Hee Lee

**Affiliations:** ^1^ Center for Integrated Nanostructure Physics (CINAP) Institute for Basic Science (IBS) Suwon 16419 Republic of Korea; ^2^ Department of Energy Science Sungkyunkwan University Suwon 16419 Republic of Korea; ^3^ School of Microelectronics Science and Technology Sun Yat‐sen University Zhuhai 519082 China; ^4^ Department of Physics Sungkyunkwan University Suwon 16419 Republic of Korea

**Keywords:** magnetism, Se‐vacancy, spintronics, two‐dimensional materials

## Abstract

The confined defects in 2D van der Waals (vdW)‐layered semiconductors can be easily tailored using charge doping, strain, or an electric field. Nevertheless, gate‐tunable magnetic order via intrinsic defects has been rarely observed to date. Herein, a gate‐tunable magnetic order via resonant Se vacancies in WSe_2_ is demonstrated. The Se‐vacancy states are probed via photocurrent measurements with gating to convert unoccupied states to partially occupied states associated with photo‐excited carrier recombination. The magneto‐photoresistance hysteresis is modulated by gating, which is consistent with the density functional calculations. The two energy levels associated with Se vacancies split with increasing laser power, owing to the robust Coulomb interaction and strong spin–orbit coupling. The findings offer a new approach for controlling the magnetic properties of defects in optoelectronic and spintronic devices using vdW‐layered semiconductors.

## Graphical abstract

The unoccupied energy levels from Se vacancy are tailored from half‐filled to full‐filled to induce the magnetic moment by modulating gate bias under field‐effect transistor in WSe_2_ semiconductors. The magneto‐photoresistance hysteresis is manifested at the resonant Se‐vacancy energy levels, implying the ferromagnetic order formation. This study opens a venue for 2D van der Waals magnetic semiconductors for future opto‐spintronics.

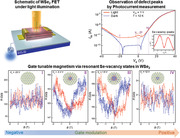

## 1 Introduction

Intrinsic defects, including vacancies and grain boundaries, are inevitably introduced as crystal imperfections formed by thermodynamic limits during synthesis. Such defects provoke deep energy levels inside the bandgap, involving carrier scattering, hence reducing the carrier mobility, which often hampers device performance.^[^ ^1^, ^2^ ^]^ Meanwhile, they can be used for single‐photon emitters and qubits in quantum information and computing.^[^ ^3^, ^4^, ^5^, ^6^, ^7^, ^8^, ^9^, ^10^ ^]^ Intrinsic metal or oxygen vacancies often trigger magnetism in oxide semiconductors and insulators.^[^ ^11^, ^12^, ^13^ ^]^ In particular, the confined defects in 2D semiconductors offer an opportunity for tailoring the electronic and magnetic properties using external forces such as strain, gate bias, electric fields, or ambient environment.^[^ ^3^, ^5^, ^14^, ^15^, ^16^, ^17^, ^18^, ^19^, ^20^, ^21^ ^]^ In vdW‐layered transition metal dichalcogenides (TMDs), intrinsic defects such as transition metals and chalcogen vacancies are often observed.^[^ ^22^, ^23^, ^24^ ^]^ Transition metal vacancies generate magnetic properties;^[^ ^25^, ^26^, ^27^ ^]^ for example, the long‐range magnetic order in PtSe_2_ is induced by the Pt vacancies inherited from the formation of occupied mid‐gap states.^[^ ^28^, ^29^, ^30^ ^]^ In contrast, the chalcogen vacancies generate unoccupied levels in the bandgap,^[^ ^31^, ^32^ ^]^ which do not contribute to the total magnetic moment. Furthermore, little is known about gate‐tunable magnetism from chalcogen vacancies in 2D semiconductors. In this study, we realized a gate‐tunable magnetic order resonant with Se vacancy states in WSe_2_ thin films by partially filling electrons. We utilized photocurrent measurements and magneto‐photoresistance hysteresis with gate sweeping using different laser powers to demonstrate gate‐tunable magnetism.

## 2 Results and Discussions

**Figure 1:**
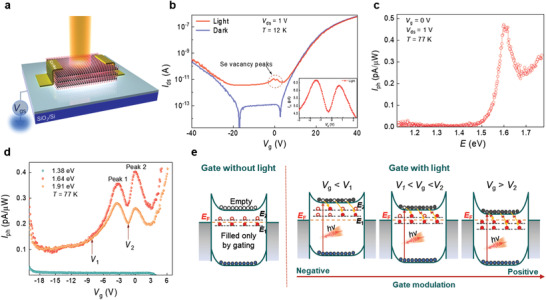
Photocurrent measurement with excitation energies under gate‐bias sweep. a) Illustration of WSe_2_ FET under white light illumination. b) *V*_g_–*I*_ds_ transfer characteristics of the WSe_2_ FET with a two‐peak feature measured at 12 K under white light illumination. The inset shows an enlarged view of the two‐peak feature (device 1). c) Photocurrent at the gate voltage of 0 V under different laser excitations at 77 K. The unit of the photocurrent is normalized by the light power density (device 2). d) Photocurrent with gate bias at different laser energies (device 2). e) Proposed mechanism of the two‐peak feature for carrier excitation and recombination.

**Figure** **1**a shows a schematic of our photocurrent measurement setup of a field‐effect transistor (FET) using a multilayer WSe_2_ channel on a SiO_2_/Si^++^ substrate. The photocurrent was measured under white light excitation in a high vacuum (10^−7^ Torr) at 12 K. A typical ambipolar behavior (slightly *n*‐doped) is observed with a high on/off current ratio of ≈10^6^ and an off‐state current of ≈10^−13^ A under the gate bias range from −20 to 5 V in the dark state (blue curve in Figure 1b). The photocurrent in the off‐state is as high as ≈10^−11^ A under white light excitation at the power of 0.3 mW (red curve). Interestingly, two separate peaks emerged notably at a gate bias between −3 and 2 V in the off‐state (inset of Figure 1b). These two peaks are related to the defect states inside the bandgap of WSe_2_. Two substantial drops in the dark current at the valence and conduction band edges originate from the charge traps at the interface between WSe_2_ and SiO_2_, which is negligible compared to the photocurrent (Figure S1, Supporting Information).

To understand the unique features of the defect states in the photocurrent measurements, different excitation energies were used to excite the carriers in the WSe_2_ channel (Figure 1c,d). The photocurrent at gate bias voltage *V*_g_ = 0 V (Figure 1c) shows a negligible response in the energy range smaller than 1.5 eV. The photocurrent begins increasing immediately after the excitation energy approaches 1.5 eV. The main peak emerges near 1.6 eV, corresponding to the vertical absorption at the K‐point in multilayer WSe_2_.^[^ ^33^ ^]^ The two‐peak feature is preserved at excitation energies of 1.64 and 1.91 eV, whereas the photocurrent is not detected in the off‐state gate‐bias with the excitation energy of 1.38 eV (Figure 1d). This implies that the photocurrent originates from the photo‐excited carriers between the conduction and valence band edges at the K point. The photocurrent at the band edge (≈1.3 eV) was negligible owing to indirect absorption below the K‐point.

The two peaks emerging with light excitation can be explained by two unoccupied energy levels located near the conduction band edge (Figure 1e). With increasing gate bias, the Fermi level shifts toward the two energy states (*E*_1_ and *E*_2_), leading to partially or fully occupied states. Nevertheless, electrons located in these states do not contribute to the electrical current flow at low temperatures owing to the limited thermal excitation. Consequently, the electrical current in the absence of light remains in the off‐state at low temperatures with the gate bias range from −20 to 5 V. Under light illumination, photo‐excited electrons and holes are generated, contributing to the photocurrent. At a relatively low gate bias (*V*_g_ < *V*_1_, where *V*_1_ is defined in Figure 1d), the trap states remain empty. The photo‐excited carriers whose excitation energy exceeds 1.6 eV will recombine to the unoccupied trap states. When *V*_1_ < *V*_g_ < *V*_2_, *E*_1_ levels are occupied, whereas *E*_2_ levels are still unoccupied. Therefore, the excited carriers recombine exclusively to the *E*_2_ levels, whereas their recombination to the occupied *E*_1_ levels is prevented, consequently increasing the photocurrent. Upon further increase in the gate bias (*V*_g_ > *V*_2_), the *E*_2_ levels start getting occupied, thereby enhancing the photocurrent further owing to the suppression of recombination to the *E*_2_ levels. The two‐peak feature disappears when the temperature exceeds ≈260 K; thus, the thermal excitation of electrons from the trap states to the conduction band edge prevails (Figure S2, Supporting Information). We note that the increase in the carrier concentration alone cannot explain the peak‐like feature or the dip *I*_ph_ with gating bias. This reduction phenomenon of *I*_ph_ originates from the change in mobility of the carriers, which reduces the current with increasing the filled defect states. Because the charged defects scatter to reduce the mobility of free carriers more dominantly than the neutral defects, the reduction of mobility becomes significant when the more neutral defects are filled with gate biases. At a certain gate bias for maximum peak 1, the effect of scattering by the charged defects is stronger than that of the increased carrier concentration, resulting in the reduction of photocurrent *I*_ph_.

**Figure 2:**
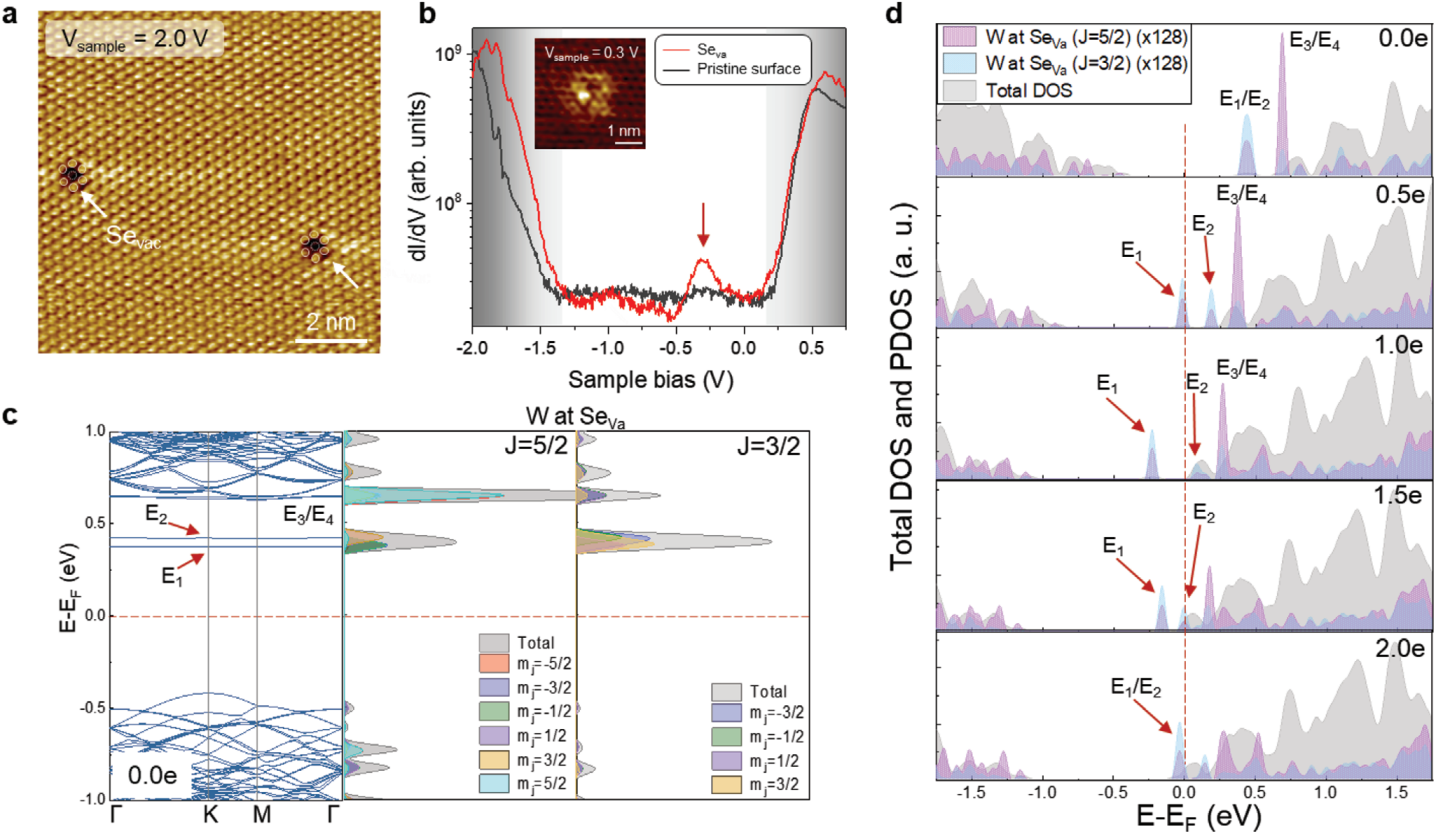
STM/STS results of Se‐vacancy states in WSe_2_ and the corresponding projected electronic density of states with charge doping. a) STM morphology of Se‐vacancies in pristine WSe_2_. b) d*I*/d*V* STS spectra measured on the position with/without Se vacancy. STM topography with Se vacancy is shown in the inset. c) DFT band structure of bilayer WSe_2_ with a Se vacancy without charge doping and the corresponding electronic density of states. d) Total and projected electronic density of states of the corresponding bilayer WSe_2_ and W near the Se‐vacancy site with different negative charges (0, 0.5, 1.0, 1.5, and 2.0 e). The defect states *E*_1_/*E*_2_ are split from each other.

To confirm the existence of trap states near the conduction band edge, we conducted scanning tunneling microscopy/spectroscopy (STM/S) measurements (**Figure** **2**). Several point defects appear as dark pits located at the Se position when the tunneling condition is *V*_sample_ = 2.0 V (Figure 2a). The bright protrusions appear as trigonal symmetry at the sample bias of 0.3 V (inset of Figure 2b), confirming the presence of Se vacancies.^[^ ^34^, ^35^, ^36^ ^]^ The STS spectrum at the Se vacancy position shows prominent Se‐vacancy‐induced mid‐gap states (Figure 2b). Meanwhile, the trap states are located below the Fermi level and hence are occupied, or the mid‐gap states are negatively charged with the *n*‐type feature in our sample. Consistent with the STM observations, a certain number of Se vacancies are visible, whereas W vacancies are not observed in scanning transmission electron microscopy measurements in different places of the WSe_2_ sample (Figure S3, Supporting Information). We note that O‐terminated Se vacancies do not contribute to the two‐peak feature in the photocurrent measurements (Figure S4, Supporting Information).

The presence of Se vacancy states in STS was confirmed through density functional theory (DFT) calculations (Figure 2c). The Se vacancy generates unoccupied *E*_1_/*E*_2_ and *E*_3_/*E*_4_ below the conduction band edge (Figure 2c and Figures S5 and S6, Supporting Information), which may be ascribed to the dangling bonds at the W site. This feature resembles the neutral S vacancies in WS_2_.^[^ ^31^, ^34^, ^35^, ^36^ ^]^ The first coupled state (*E*_1_/*E*_2_) is attributed to both the *J* = 3/2 and *J* = 5/2 states of the d orbital of W, whereas the second one (*E*_3_/*E*_4_) originates primarily from *J* = 5/2, with a small contribution from *J* = 3/2 (Figure S6, Supporting Information). The distinct peaks between *E*_1_/*E*_2_ and *E*_3_/*E*_4_ from the DFT calculations do not originate from the two trap states of *E*_1_ and *E*_2_, according to the photocurrent measurements (Figure 1) or STM/STS (Figure 2b). In fact, the seemingly degenerate *E*_1_/*E*_2_ peak can be split into two peaks due to bonding–antibonding states by partially filling the charges (0.5 e). The split energy further increases from 0.2 to 0.3 eV when the charge is increased from 0.5 to 1.0 e. At 1.5 and 2.0 e, the bonding–antibonding states are converted to the bonding *E*_2_ state, reducing the repulsion between *E*_1_ and *E*_2_. The variation of the *E*_1_ and *E*_2_ states with charge injection was congruent with the two prominent peaks in the photocurrent measurements. Notably, the coupled *E*_3_/*E*_4_ moves further inside the conduction band as the negative charge increases. This cannot be observed in our gate‐photocurrent and STM measurement results.

**Figure 3:**
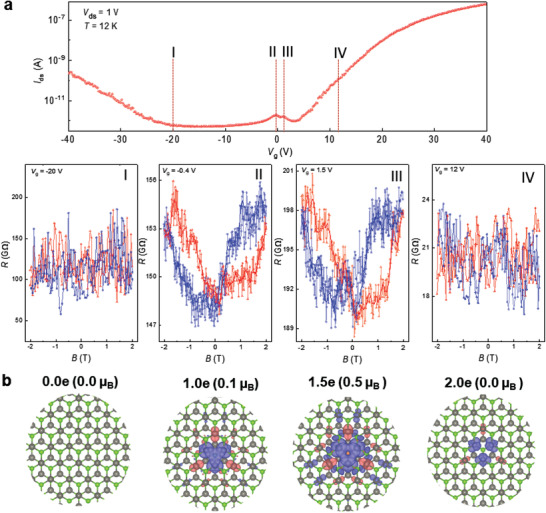
Magnetic order in energy levels with Se‐vacancy in WSe_2_ (device 1). a) *V*_g_–I_ds_ transfer curve (top panel) and magnetoresistance measurement with different gate biases at 12 K (bottom panel). In the magnetoresistance curves (bottom panel), a full cycle of magnetic field (−2T to +2T) was scanned with a forward sweeping from −2T to +2T (blue curve) and a reverse sweeping from +2T to −2T (red curve). Magnetic hysteresis appears at II and III gate biases. b) Spin density at different negative charge states highlighted in adjacent Se vacancy (bottom panel). The number of charges is different from the calculated total magnetic moment. The spin density indicates the significant contribution of delocalized states together with the localized states near Se‐vacancies. The isosurface value is 0.0002.

To determine the possible magnetic order in gate‐tunable Se vacancy states in WSe_2_, we measured the magnetoresistance with gate bias under light excitation at 12 K with a full cycle of the magnetic field from −2 to +2 T (**Figure** **3**a). When the gate bias *V*_g_ << *V*_peak1_ (the Fermi level lies above the valence band edge) or *V*_g_ >> *V*_peak2_ (Fermi level lies below the conduction band edge), the magneto‐photoresistance hysteresis is almost negligible during the forward and backward sweeps of the magnetic field (I and IV). Interestingly, the magneto‐photoresistance hysteresis is clearly manifested at the resonant energy levels within the bandgap matched with the gate bias at the peak positions (II and III), implying the formation of a magnetic order.

In fact, a local magnetic moment is formed at W atoms near Se vacancies when two energy levels are partially occupied at the gate biases of II and III. Furthermore, the spin density distribution from the DFT calculations is illustrated around the Se vacancies near the W sites with different charge injections or, equivalently, gate bias in Figure 3b. Spin density is absent without injecting charge (I), which corresponds to the absence of magnetoresistance hysteresis with the magnetic field when *V*_g_ << *V*_peak1_. With increasing electron injections (II and III), the up‐spin density accumulates more in the W sites near the Se vacancies. Consequently, the magnetic moment is apparently promoted in the partially occupied energy levels, which is consistent with the observed magnetoresistance hysteresis in Figure 3a. With further electron injection (or high positive gate bias), the total magnetic moment (sum of the spin density of the supercell) becomes zero despite the presence of the up/down‐spin density near the Se vacancies, consistent with negligible hysteresis when *V*_g_ >> *V*_peak2_. We note that the total magnetic moment is not the same as the number of charges, partially owing to the wide distribution of spins in the lattice. The presence of partially occupied defect states in TMDs, particularly the Se vacancies in WSe_2_, plays a key role in the creation of magnetic order.

**Figure 4:**
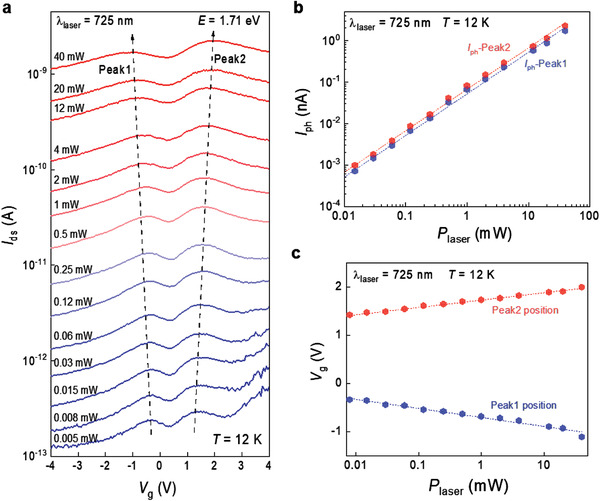
Laser‐power‐dependent variations of *E*_1_ and *E*_2_ (device 3). a) Laser‐power‐dependent photocurrent with gate biases. Peak 1 and peak 2 are separated. The dashed arrows indicate the shift of the two peaks. b) Photocurrents with different laser powers (log–log scale) at two defect states. c) Change in two‐peak positions for different laser powers (local maximum photocurrents with gate biases in [a]).

We further investigated the splitting states of *E*_1_ and *E*_2_ with laser power dependence (**Figure** **4**a and Figure S7, Supporting Information). As the laser power increased, the photocurrent was enhanced with two prominent peaks of *E*_1_ and *E*_2_; more importantly, the *E*_1_ peak position in the photocurrent is downshifted with gate bias, whereas the *E*_2_ peak position is upshifted. The maximum peak intensity of each peak position (Figure 4b) is well fitted using the power‐law *I*_ph_ = *bP^*α*^*, where *b* is a proportionality constant and *α* is a dimensionless exponent.^[^ ^37^, ^38^, ^39^ ^]^ In contrast with *α* < 1, which indicates the presence of trap states for minority carriers, *α* is ≈1 in our sample, implying a trap state for majority carriers. As the power increases, the peak 1 (*E*_1_) near *V*_g_ = −0.5 V shifts toward the negative bias, whereas peak 2 (*E*_2_) shifts toward the positive bias (Figure 4c).

The two gate biases corresponding to the *E*_1_ and *E*_2_ positions are separated from each other with increasing laser power. This phenomenon can be explained by the strong Coulomb repulsion combined with the spin–orbit coupling (SOC). While the Coulomb interaction is still strong in 2D materials with separated *E*_1_ and *E*_2_ peaks even without SOC, the separation of the two peaks becomes prevalent with SOC (Figure S8, Supporting Information). The number of spins trapped in the two energy levels of *E*_1_ and *E*_2_ resonant by the gate bias accumulates with increasing photo‐excited carriers. Consequently, the spin–spin repulsion becomes more prominent with increasing laser power.

The appearance of Se‐vacancy spin states near the conduction band edge in WSe_2_ is analogous to the shallow donor impurity band in diluted ferromagnetic oxides.^[^ ^36^, ^40^ ^]^ In such materials, long‐range magnetic order can be established by magnetic polarons, which are formed by exchange interactions between the local spins at trap states and free spin carriers.

In our study, the thickness of WSe_2_ is ≈5–10 nm. Thick layers are used to self‐protect Se vacancies from ambient gases during device fabrication and characterization. This ensures to investigate the properties of the intrinsic Se vacancies. The bandgap shrinks as the thickness of WSe_2_ increases. Since the trend of photocurrent measurements is not appreciable with slightly different thicknesses, the energy difference between the conduction band edge and the defect levels is presumably negligible. The appearance of magnetism from defects with gate bias at high temperature strongly relies on the nature of materials: Large‐bandgap materials such as MoS_2_ and WS_2_, particularly in the monolayer form could be examples. In such a large bandgap system, the defect states induced by S vacancies are presumably far from the conduction band edges compared to Se vacancies in MoSe_2_ and WSe_2_. The Curie temperature can be modulated by tuning the vacancy concentration. This requires further investigation to demonstrate gate‐tunable spintronic devices with magnetic heterostructures.

## 3 Conclusion

In summary, we successfully demonstrated a gate‐tunable magnetic order in pristine WSe_2_ by tuning the occupation of the Se vacancy states. The strong Coulomb repulsion together with the SOC associated with the defect states of Se vacancies separates spin states with carrier doping, leading to the formation of magnetism by hybridizing the localized spin states with free carriers. We propose the possibility of engineering defects in vdW‐layered materials for magneto‐optoelectronics and gate‐tunable spintronic devices.

## Conflict of Interest

The authors declare no conflict of interest.

## Author Contributions

T.D.N., J.J., and B.S. contributed equally to this work. T.D.N., J.J., and D.L.D. initiated this work. T.D.N. and J.J. fabricated and characterized FET devices. B.S. measured and analyzed STM/S data. M.D.T., T.D.N., J.J., and J.H.K. performed wavelength‐dependent photocurrent measurement and its analysis. W.C. and Y.‐M.K. performed TEM measurement and analysis. D.L.D. performed DFT simulation. D.L.D. and Y.H.L. guided and analyzed the work. All authors participated in the discussion and the manuscript preparation.

## Supporting information

Supporting Information

## Data Availability

All data are available in the main text or the Supporting Information.
