# Supplementary material for: Gate‐Tunable Magnetism via Resonant Se‐Vacancy Levels in WSe2
Source: Adv Sci (Weinh). 2021 Oct 28;8(24):2102911. doi: 10.1002/advs.202102911 (PMC8693072; doi:10.1002/advs.202102911)
Supplement: Supplementary file 1 — Supporting Information [file ADVS-8-2102911-s001.pdf]

© 2021 Wiley-VCH GmbH

ADVANCED SCIENCE
Open Access

Supporting Information

for *Adv. Sci.*, DOI: 10.1002/advs.202102911

Gate-tunable magnetism via resonant Se-vacancy levels in WSe_2

Tuan Dung Nguyen[1,2†], Jinbao Jiang[1,3†], Bumsub Song[1,2†], Minh Dao Tran[1], Wooseon Choi[1,2], Ji Hee Kim[1,2], Young-Min Kim[1,2], Dinh Loc Duong[1,2], Young Hee Lee[1,2,4*]*

Supporting Information

Gate-tunable magnetism via resonant Se-vacancy levels in WSe_2

Tuan Dung Nguyen, Jinbao Jiang, Bumsub Song, Minh Dao Tran, Wooseon Choi, Ji Hee Kim, Young-Min Kim, Dinh Loc Duong, Young Hee Lee**

EXPERIMENTAL SECTION

Sample Preparation

A single crystal of WSe_2 was synthesized by chemical vapor transport (CVT). The reactant powders of high-purity tungsten (99.999%, Sigma Aldrich) and selenium (99.998%, Sigma Aldrich) were mixed and ground uniformly at a nominal ratio. The mixed powder was compressed into a pellet before loading into quartz ampoules for sealing under high vacuum and annealing at 1000 °C for 24 h. The polycrystalline powder was collected and resealed after adding iodine as a transporting agent. The temperatures of the cold and hot ends were 935 °C and 1050 °C, respectively. The entire growth time was prolonged to 12 d.

Device Fabrication and Characterization

Device Fabrication: Few-layered pristine WSe_2 flakes were exfoliated onto a SiO_2/Si substrate using Scotch tape. The samples were then spin-coated using polymethyl methacrylate, and the substrate was heated on a hot plate at 160 °C for three min. Finally, the source (S) and drain (D) contacts (Cr/Au of 5/50 nm) were patterned by e-beam lithography before metal deposition.

Device Characterization: Photocurrent measurements were performed under white light excitation using an optical halogen lamp (EJV-KLS Japan) in high vacuum (~10^{-7} Torr) at 12 K. Electrical characterizations and magnetoresistance measurements were performed under high vacuum (~10^{-7} Torr) with a Hall-probe station system (Lake Shore Cryotronics system CRX-VF) and semiconductor analyzer (Keithley 4200 system).

Wavelength-dependent photocurrent measurement

The device was illuminated using monochromatic light, which was extracted from a white-light source (EQ-99FC LDLS) passing through a monochromator (PI Acton SpectraPro SP-2155). The spectral response was measured with a series of solid-state lasers combined with a monochromator, which was collimated and focused onto the samples in the vacuum chamber using an objective lens. The photocurrent of the device was acquired using a current preamplifier

(Femto DLPCA-200) together with a Stanford Research SR 830 lock-in amplifier and an analyzer (SMU Keithley 4200 system) for the gate biases. The photocurrent spectrum was normalized to the power spectrum of the supercontinuum light source. The two-peak features are repeatedly observed in our devices, although the peak positions differ with variations in defect densities and thicknesses of the samples. The data in Figure 1b, Figure 1c, d, and Figure 3, 4 were collected from the corresponding three different devices.

Scanning tunneling microscopy/spectroscopy (STM/S) and Scanning transmission electron microscopy (STEM)

STM/S was performed in an ultrahigh vacuum chamber with a base pressure of ~10^{-11} Torr using a commercial VT-STM (Omicron, Germany). Electrochemically etched W tips were used after the removal of surface oxides by electron bombardment in an ultrahigh vacuum chamber. The tips were calibrated by measuring the reference spectra on the highly oriented pyrolytic graphite (HOPG) substrate to avoid tip artifacts. A tunneling bias was applied to the samples. Prior to scanning, the WSe_2 sample was cleaved and subsequently annealed in an ultra-high vacuum chamber (~10^{-11} Torr) at ~500 °C for 30 min. During the STS measurements, the conventional lock-in technique with a voltage modulation of 36-50 mV_{rms} at 919 Hz was used. To measure Se vacancy content in the WSe_2 sample, annular dark-field (ADF) STEM images were taken by a probe aberration-corrected JEM ARM 200F instrument operating at 80 keV with a nominal probe current of 25 pA. The angle range of ADF detector was set to be ~68–280 mrad.

Density functional theory (DFT) calculations

DFT calculations were performed using the generalized-gradient approximation with the Quantum Expresso package[1]. To isolate the Se-vacancy, a supercell of 8 × 8 was used for the calculations, with 3× 3 × 1 and 2 × 2 × 1 k-point grids for the monolayer and bilayer, respectively. The projector augmented potential approach[2] with an energy cut-off of 400 eV was used. The total energy of the system was self-consistently calculated with a convergence of 10^{-5} eV and smearing value of 0.001 Ry. The geometry of the defect was optimized until the force was smaller than 10^{-3} Hartree/Bohr. A U value of 2.5 was used for W, which was determined by fitting the separation energy between E_1/E_2 and E_3/E_4 with experimental observations in WS_2[3,4] (Figure S5).

SUPPLEMENTARY FIGURES

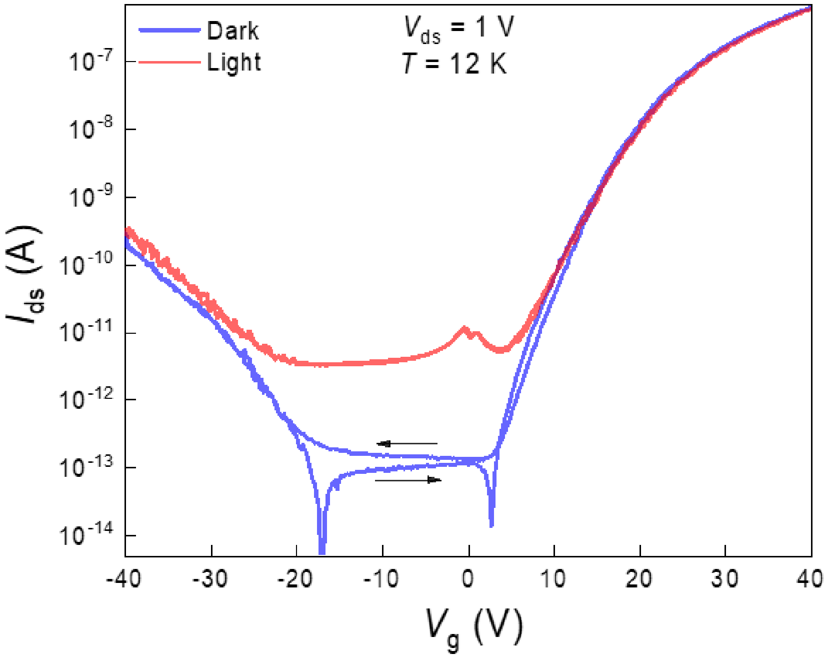

Figure S1. V_g-I_{ds} transfer characteristics of the WSe_2 FET under dual sweep of gate biases in dark state (blue line) and under light illumination (red line). Data was measured using the device 1.

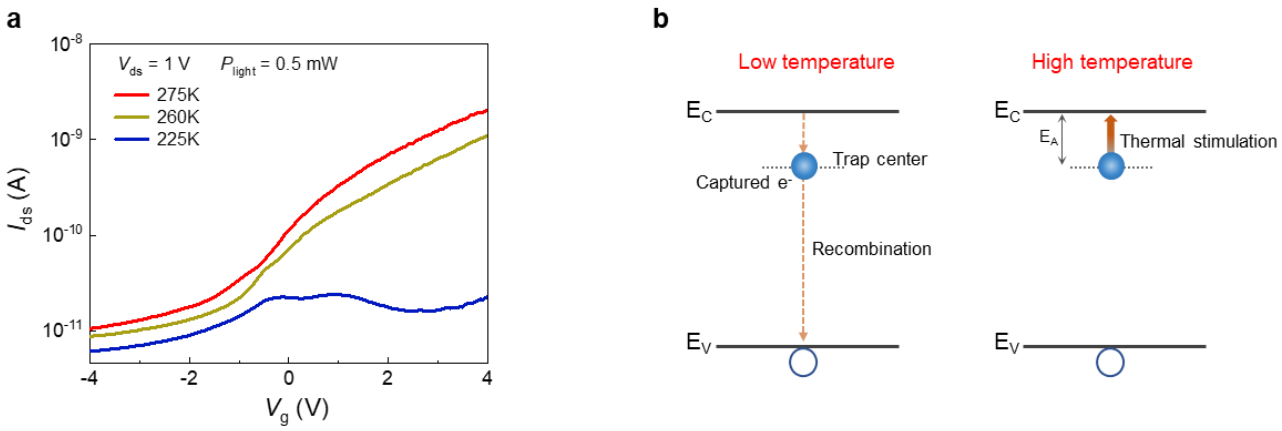

Figure S2. Temperature-dependent photocurrent measurement. (a) Temperature-dependent photocurrent measurement under a light power of 0.5 mW. Two defect peaks were maintained at 225 K. These peaks disappear when the temperature exceeds 260 K. (b) Schematic of the thermal excitation of electrons located in the trap sites at high temperature. At high temperatures, the electrons captured into the trap states receive sufficient thermal energy to be excited to the conduction band[5–7]. Data was measured using the device number 3.

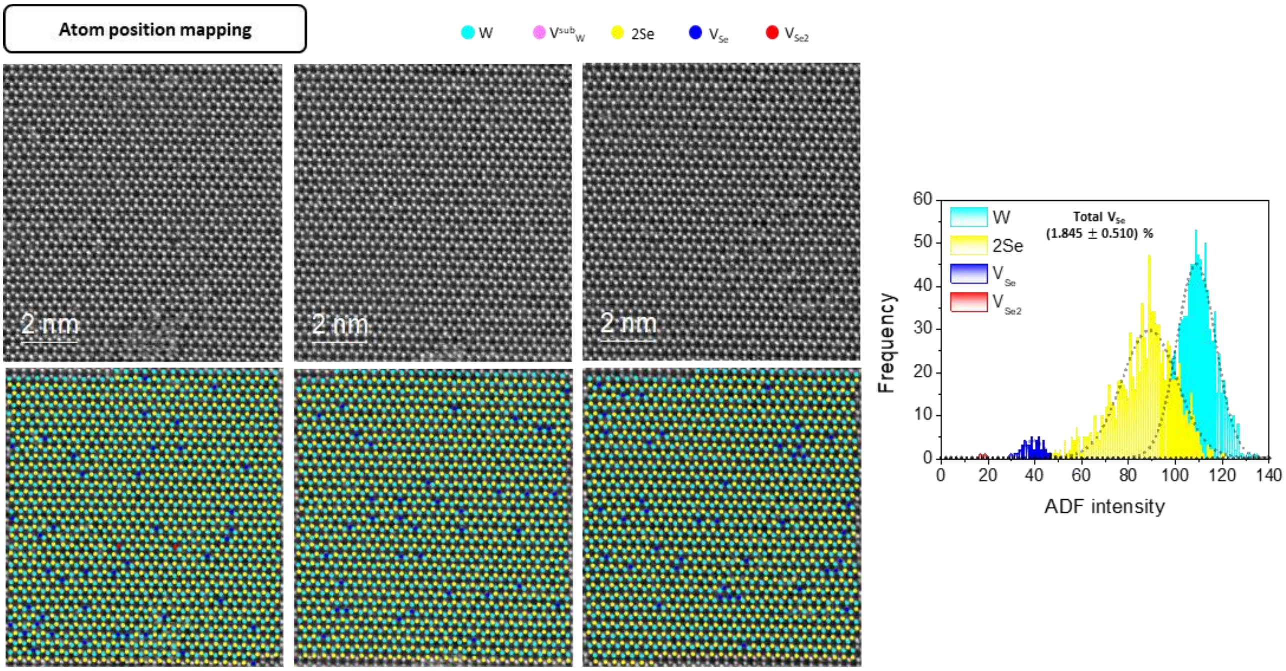

Figure S3. STEM observations of Se vacancies in pristine WSe_2. Single (solid blue dots) and double Se vacancies (solid red dots) were detected *via* different probing areas. The contrast in annular dark-field scanning transmission electron microscopy (ADF-STEM) was analyzed using the column intensity histogram. From the histogram of the ADF signal intensity, the total density of Se vacancies is calculated by approximately 1.85%. As the samples were exposed under ambient conditions during the STEM measurements, the Se-vacancy sites could have been terminated by oxygen atoms, which were indistinguishable in the STEM observations[3].

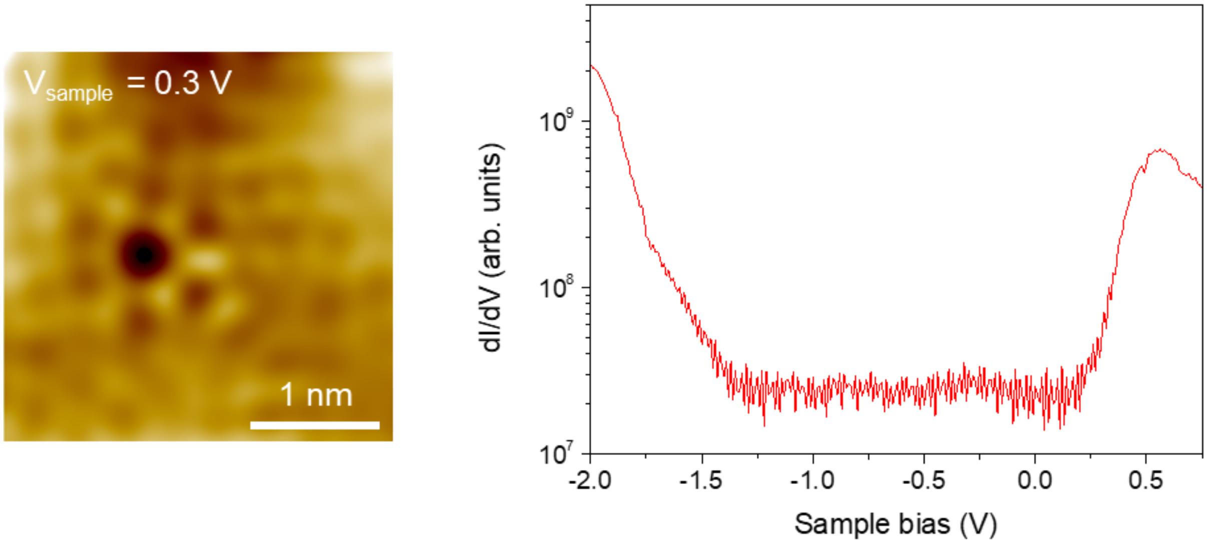

Figure S4. STM measurements of O-terminated Se vacancies. At a sample bias of 0.3 V, O-terminated Se vacancies show a dark pit with a trigonal symmetry, similar those reported earlier[3,4]. Interestingly, the d*I*/d*V* spectrum acquired at this type of defect exhibits no spectroscopic features that differ from that of the pristine surface. This is attributed to the fact that the isoelectric properties of O and Se result in the passivation of the shallow states arising from the Se vacancies.

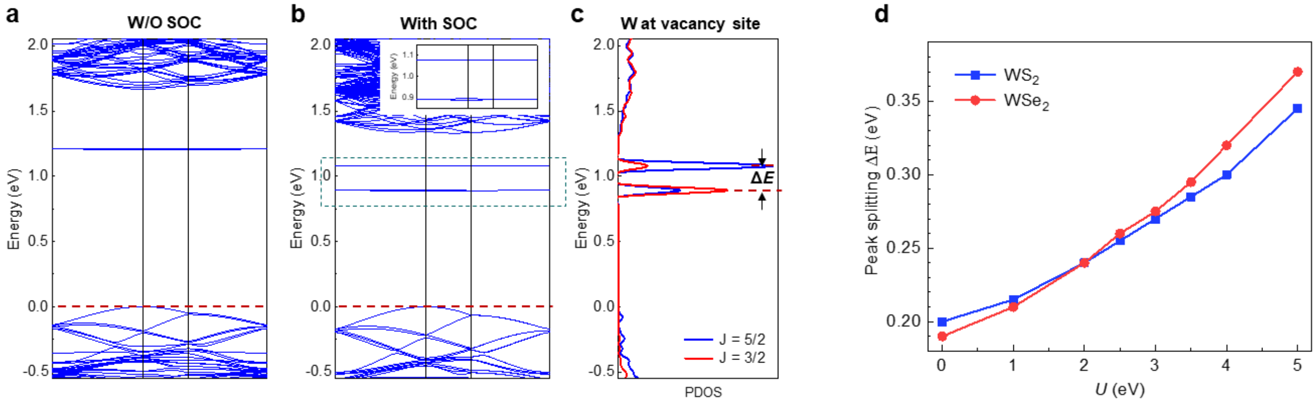

Figure S5. Determining the U value of W by fitting the experimental data of WS_2 in a previous report[4]**.** Band structures of the Se-vacancy WS_2 monolayer without (a) and with (b) spin-orbit coupling (SOC). The SOC splits the degenerate defect states into two pairs of states E_1/E_2 and E_3/E_4. The projected DOS of the W atoms at the vacancy sites reveals their significant contribution to these states. (c) The low-energy coupled state is attributed to J=3/2 and J=5/2 of the d orbital of W whereas the high-energy state mainly originates from J=5/2 with a small contribution of J=3/2. The energy gap between these two couples is 200 meV. It is to be noted that the experimental separation of these two peaks is 250 meV[4]. GW calculations yields the value of 200 meV for this energy gap although the bandgap is overestimated[4]. To understand the physical origin of large energy separation between these two states, the GGA+U method was used, as shown in Figure S4d. Higher values of U correspond to larger separations. When U=2.5, the energy separation is ~250 meV, consistent with the experimental value for WS_2[4] (d). As the strong Coulomb interaction is associated with the d-orbital of W, we apply U = 2.5 to calculate the band structure of the Se-vacancy WSe_2 monolayer.

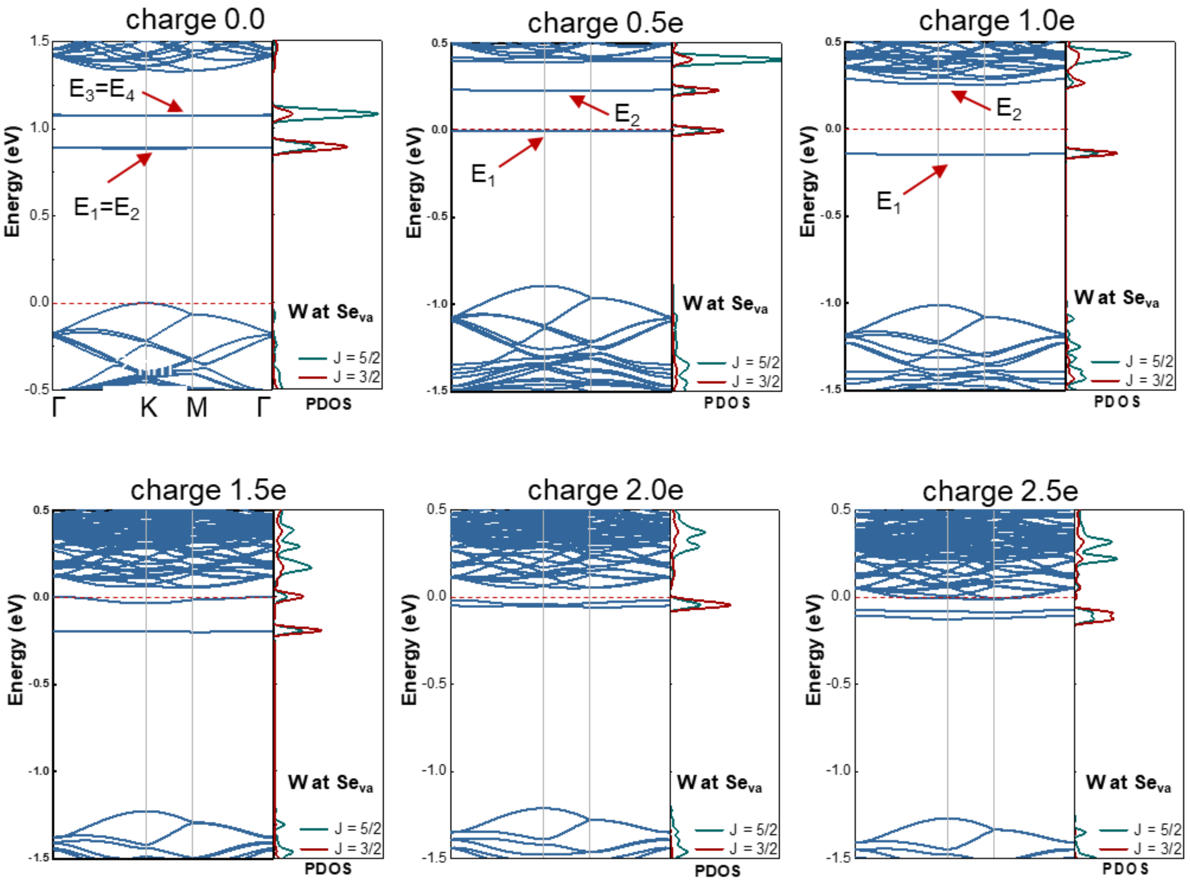

Figure S6. DFT band structure with different negative charges (0.5 e, 1.0 e, 1.5 e, 2.0 e and 2.5 e) in the WSe_2 monolayer with an Se vacancy. The degenerate defect states E_1/E_2 are split into two separated states owing to the strong Coulomb interaction. The splitting of the E_1/E_2 in the monolayer is consistent with that in the bilayer. The E_1 and $E_{2,}$ which are slightly split in the bilayer, are almost degenerate in the monolayer without charge doping (Figure 2c). In addition, the E_3/E_4 states are located closer to the conduction band edge of the bilayer than that of the monolayer owing to the smaller bandgap of the bilayer (Figure 2c). We note that the E_3/E_4 pair moves further inside the conduction band as the negative charge increases and thus, cannot be observed inside the bandgap if the Se vacancies are in the negatively charged state in our gate-photocurrent and STM measurements. The mid-gap state observed in the STM may be related to either the single peak E_1 or double peak E_1/E_2 in the corresponding 1e or 2e charge states.

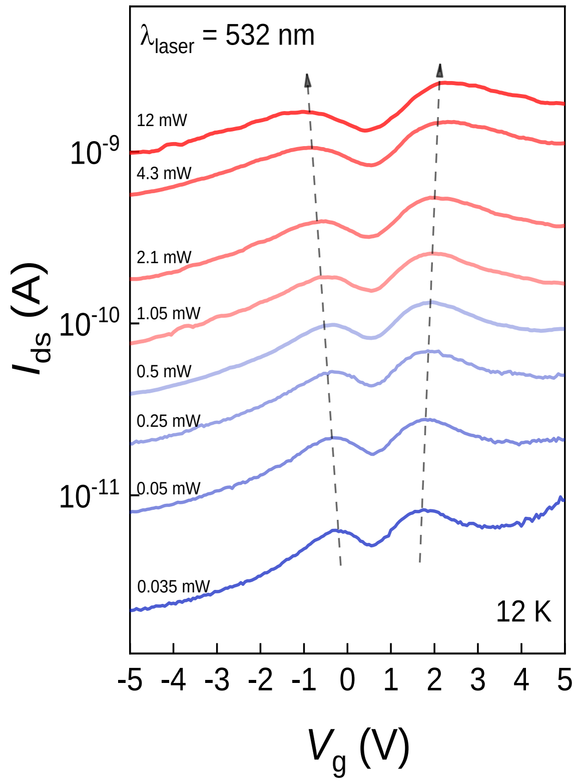

Figure S7. Gate-sweep dependent photocurrent as a function of the laser power (under 532 nm laser). Data was measured using the device number 3.

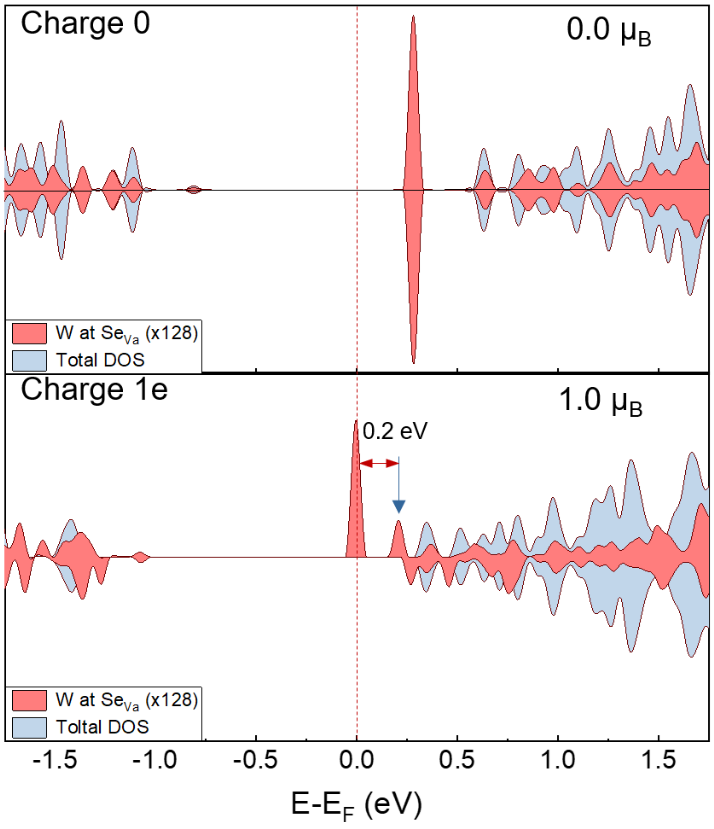

Figure S8. Electronic band structure of the WSe_2 bilayer with one Se vacancy without spin-orbit coupling (SOC) at charge 0 and 1 e. In the uncharged state, the total density of states of spin-up and spin-down are equal, implying the absence of a magnetic moment of the Se vacancy. Nevertheless, a magnetic moment of 1.0 μ_B is induced by a charge of 1 e. Interestingly, spin-spin splitting appears with electron doping at the Se-vacancy states, which is approximately 0.2 eV. This value is further enhanced up to 0.3 eV with SOC (Figure 2d).

REFERENCE

1. P. Giannozzi, O. Andreussi, T. Brumme, O. Bunau, M. Buongiorno Nardelli, M. Calandra, R. Car, C. Cavazzoni, D. Ceresoli, M. Cococcioni, N. Colonna, I. Carnimeo, A. Dal Corso, S. de Gironcoli, P. Delugas, R. A. DiStasio, A. Ferretti, A. Floris, G. Fratesi, G. Fugallo, R. Gebauer, U. Gerstmann, F. Giustino, T. Gorni, J. Jia, M. Kawamura, H.-Y. Ko, A. Kokalj, E. Küçükbenli, M. Lazzeri, M. Marsili, N. Marzari, F. Mauri, N. L. Nguyen, H.-V. Nguyen, A. Otero-de-la-Roza, L. Paulatto, S. Poncé, D. Rocca, R. Sabatini, B. Santra, M. Schlipf, A. P. Seitsonen, A. Smogunov, I. Timrov, T. Thonhauser, P. Umari, N. Vast, X. Wu, S. Baroni, Advanced capabilities for materials modelling with Quantum ESPRESSO. *J. Phys. Condens. Matter*. **29**, 465901 (2017).
2. E. Kucukbenli, M. Monni, B. I. Adetunji, X. Ge, G. a Adebayo, N. Marzari, S. de Gironcoli, a D. Corso, Projector augmented-wave and all-electron calculations across the periodic table: a comparison of structural and energetic properties.
3. S. Barja, S. Refaely-Abramson, B. Schuler, D. Y. Qiu, A. Pulkin, S. Wickenburg, H. Ryu, M. M. Ugeda, C. Kastl, C. Chen, C. Hwang, A. Schwartzberg, S. Aloni, S.-K. Mo, D. Frank Ogletree, M. F. Crommie, O. V. Yazyev, S. G. Louie, J. B. Neaton, A. Weber-Bargioni, Identifying substitutional oxygen as a prolific point defect in monolayer transition metal dichalcogenides. *Nat. Commun.* **10**, 3382 (2019).
4. B. Schuler, D. Y. Qiu, S. Refaely-Abramson, C. Kastl, C. T. Chen, S. Barja, R. J. Koch, D. F. Ogletree, S. Aloni, A. M. Schwartzberg, J. B. Neaton, S. G. Louie, A. Weber-Bargioni, Large Spin-Orbit Splitting of Deep In-Gap Defect States of Engineered Sulfur Vacancies in Monolayer WS_2. *Phys. Rev. Lett.* **123**, 076801 (2019).
5. S. O. Kasap, *Principles of Electronic Materials & Devices* (2018).
6. J. Singh, *Semiconductor optoelectronics: physics and technology* (1995).
7. M. M. Islam, D. Rana, A. Hernandez, M. Haseman, F. A. Selim, Study of trap levels in β-Ga_2O_3 by thermoluminescence spectroscopy. *J. Appl. Phys.* **125** (2019).
